# The Scavenging of DPPH, Galvinoxyl and ABTS Radicals by Imine Analogs of Resveratrol

**DOI:** 10.3390/molecules21010127

**Published:** 2016-01-21

**Authors:** Peter Kotora, František Šeršeň, Juraj Filo, Dušan Loos, Juraj Gregáň, Fridrich Gregáň

**Affiliations:** 1 Institute of Chemistry, Faculty of Natural Sciences, Comenius University in Bratislava, Mlynská dolina, 842 15 Bratislava, Slovakia; f.sersen@gmail.com (F.Š.); filo@fns.uniba.sk (J.F.); dusan.looos@chello.sk (D.L.); 2 Department of Genetics, Faculty of Natural Sciences, Comenius University in Bratislava, Mlynská dolina, 842 15 Bratislava, Slovakia; 3 Department of Chromosome Biology, MFPL, University of Vienna, Dr. Bohr-Gasse 9, 1030 Vienna, Austria; 4 Department of Chemistry, Faculty of Natural Sciences, Matej Bell University, Tajovského 40, 974 01 Banská Bystrica, Slovakia; Fridrich.Gregan@umb.sk

**Keywords:** resveratrol analogs, iminophenols, antioxidant activity

## Abstract

Resveratrol (3,5,4′-trihydroxystilbene) is a phytoalexin produced by plants. Resveratrol is known for its anti-cancer, antiviral and antioxidant properties. We prepared imine analogs of resveratrol ((hydroxyphenyliminomethyl)phenols) and tested their antioxidant activity. All prepared resveratrol analogs were able to scavenge 2,2-diphenyl-1-picrylhydrazyl (DPPH), galvinoxyl radical (GOR) and 2,2′-azino-bis(3-ethylbenzothiazoline)-6-sulphonic acid (ABTS) radicals. The antioxidant activity efficiency correlated with the number and position of hydroxyl groups. The most effective antioxidants were resveratrol analogs containing three hydroxyl groups in the benzylidene part of their molecules. These results provide new insights into the relationship between the chemical structure and biological activity of resveratrol analogs.

## 1. Introduction

**Figure 1:**
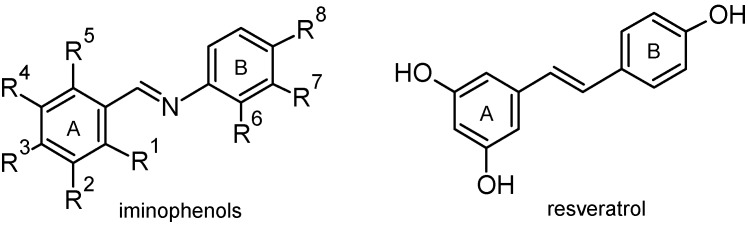
Structure of resveratrol and studied Hydroxyphenyliminomethyl)phenols.

The structure of (hydroxyphenyliminomethyl)phenols (Figure 1) is similar to the structure of resveratrol (3,5,4′-trihydroxystilbene), which exhibits various biological activities. There are two geometric isomers of resveratrol—the *trans* configuration undergoes isomerisation to the *cis* form following exposure to ultraviolet light. Resveratrol is a phytoalexin produced by several plants that was initially identified in the flowering plant *Veratrum grandiflorum*. Relatively high concentrations of resveratrol are also found in grape skins and seeds, as well as red wine. Resveratrol has gained widespread attention due to its ability to extend the lifespan of various organisms, to protect against age-related diseases such as cancer and a broad range of biological activities including antiviral and antioxidant properties [1,2,3,4]. However, it is not clear whether the health benefits associated with wine drinking (“French paradox”, the observation of reduced incidence of cardiovascular disease in regions of France where red wine and fats are consumed in large quantities) are due to resveratrol [5]. Molecular mechanisms underlying the health-enhancing effects of resveratrol are not well understood but some of them may be due to its antioxidant properties [6]. Although basal level of oxidative stress is important for normal cell function, severe oxidative stress often leads to oxidative damage, cell death and various diseases [7]. Resveratrol supplementation decreases the oxidative stress caused by consumption of a high calorie meal [8]. In addition, resveratrol is a potent activator of SIRT1, member of sirtuin family of protein deacetylases implicated in counteracting various age-related diseases [9].

Previous studies showed that modification of resveratrol could enhance its biological activities [10,11,12]. It is therefore important to synthesize resveratrol analogs and study their physico-chemical and biological properties. Imines are often used as ligands for the complexation of metals; they are used for the preparation of liquid crystals and are also used in analytical and pharmaceutical chemistry [13]. The traditional method of the imine preparation is based on heating aldehydes with amines in an organic solvent (benzene or toluene) [13,14,15]. Synthesis in which the organic solvent is replaced with water [16] or other recycle medium such as polypropylene glycol [17] has been described as “green” alternative to these preparations.

The goal of this work was to prepare various imine analogs of resveratrol (some of which have not been described in the literature yet) and to test their antioxidant activity. We analyzed antioxidant properties of twenty-one (hydroxyphenyliminomethyl)phenols (Figure 1) where R^i^ = OH or H.

The studied (hydroxyphenyliminomethyl)phenols consist of two parts; one is aminophenol (AP) and the other is phenolic part from hydroxybenzaldehyde (HBA). Some of these compounds are produced as secondary metabolites in plants. 4-HBA and 3,4-dihydroxy-benzaldehyde (DHBA) are present in *Origanum vulgare* L. [18], oil palm biomass [19], *Potentilla fulgens* [20], sugarcane molasses [21] and vanilla pods [22]. 4-HBA and 4-AP are present in *Dilobeia thouarsii* [23]. Many HBAs are known for their flavoring and antimicrobial properties [24,25]. Antioxidant properties of some HBAs (2-HBA, 3-HBA, 3,4-DHBA) were studied by the Tsimidou group [26,27]. Some of imine resveratrol anologs were studied as multifunctional drugs in the treatment of Alzheimer’s dissease [28] and they also exhibit antioxidant activity [28,29]. Some hydroxybenzene diimine derivatives possess important biological activities such as antioxidant [30], antiinflammatory and anti-analgesics [31].

## 2. Results and Discussion

### 2.1. Chemistry

**Scheme 1:**
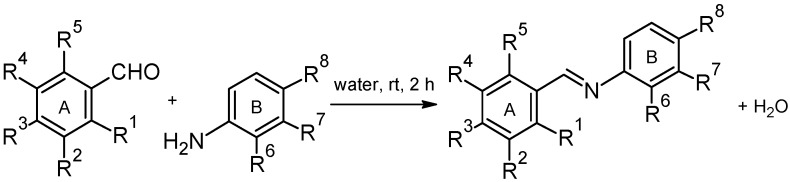
Preparation of iminophenol analogs of resveratrol.

The starting compounds for preparation of (hydroxyphenyliminomethyl)phenols were mono aminophenols (APs) and mono-, di- and trihydroxybenzaldehydes (HBAs). Twenty-one (hydroxyphenyliminomethyl)phenols (compounds **1**–**21**, for full names see Section 3.2. Synthesis) were prepared by condensation reactions where the first step was addition of amino group on carbonyl group followed by elimination of water according to protocols of Tanaka and Shiraishi [16] and Lu *et al.* [29] (Scheme 1). Briefly, equimolar amounts of appropriate AP and HBA were stirred in a small amount of distilled water at room temperature (25 °C) for 2 h. Obtained products were filtered, washed several times with distilled water and dried at 45 °C in vacuum. Yields of (hydroxyphenyliminomethyl)phenols ranged between 26% and 95%. The compounds were characterized by melting point, elemental analysis, ^1^H- and ^13^C-NMR, IR and MS spectra.

### 2.2. Antioxidant Activity

All prepared (hydroxyphenyliminomethyl)phenols **1**–**21** were able to scavenge 2,2-diphenyl-1-picrylhydrazyl (DPPH), galvinoxyl and 2,2′-azino-bis(3-ethylbenzothiazoline)-6-sulphonic acid (ABTS) radicals (Table 1). This table shows that some of the prepared (hydroxyphenyliminomethyl)phenols were more effective scavengers of DPPH (compounds: **8**, **10**, **12**, **14**, **17**–**21**) or galvinoxyl (GOR) (compounds **2**, **9**, **13**, **16**, **17**–**21**) as compared to resveratrol (Table 1). On the other hand all prepared (hydroxyphenyliminomethyl)phenols exhibited lower efficiency of ABTS scavenging as resveratrol, their SC_50_ values ranged from 1.98 to 18.16 μmol/dm^3^.

**Table 1:** SC_50_ and proton affinity (PA) + electron transfer enthalpy (ETE) values of studied (hydroxyphenyliminomethyl)phenols.

Compound	DPPH SC_50_/r^2^ (μmol/dm^3^)	GOR SC_50_/r^2^ (μmol/dm^3^)	ABTS SC_50_/r^2^ (μmol/dm^3^)	PA + ETE in Methanol (kJ/mol)	PA + ETE in Water (kJ/mol)
**1**	27.90/0.951	184/0.985	11.64/0.969	546.8	562.2
**2**	38.26/0.963	48.27/0.998	8.50/0.809	550.9	566.0
**3**	560/0.893	3075/0.809	8.50/0.928	547.7	562.2
**4**	967/0.976	415/0.995	3.54/0.993	565.4	580.7
**5**	383/0.936	393/0.903	6.74/0.869	547.1	562.1
**6**	88.64/0.974	251/0.999	3.86/0.994	550.1	564.4
**7**	53.98/0.937	184/0.979	6.57/954	547.4	562.8
**8**	21.00/0.997	127/0.971	14.39/0.972	543.1	551.9
**9**	43.00/0.991	39.96/0.945	18.16/0.922	520.8	537.4
**10**	19.00/0.994	2300/0.987	6.4/0.988	539.6	554.7
**11**	83.00/0.947	73.78/0.998	3.05/0.847	554.6	569.9
**12**	24.00/0.970	456/0.995	3.31/0.968	541.8	554.0
**13**	18.00/0.988	23.34/0.997	6.40/0.977	542.7	557.6
**14**	12.52/0.964	102/0.865	5.83/0.988	547.5	573.9
**15**	42.00/0.986	55.43/0.727	3.74/0.918	540.3	555.4
**16**	149/0.979	173/0.988	2.53/0.962	554.9	570.2
**17**	22.05/0.988	25.24/0.989	2.01/0.989	537.4	552.7
**18**	12.60/0.996	27.67/0.993	2.83/0.956	525.8	540.7
**19**	9.05/0.988	72.10/0.958	2.46/0.977	531.1	546.5
**20**	18.05/0.988	23.92/0.985	2.92/0.9997	548.4	563.1
**21**	8.77/0.943	15.39/0.994	1.98/0.994	528.5	544.0
Resveratrol	26.37/0.849	72.66/0.910	1.43/0.959	548.6	563.5

r^2^ is an average square deviation.

Antioxidant activities of compounds **1**, **2**, **3**, **4**, **5**, **7**, **9** and **16** have already been studied [28,29]. SC_50_ values reported by Lu *et al.* [29] and Li *et al.* [28] are similar to those presented in our current study. However, our melting points NMR spectra of compounds **2** and **9** do not correspond with those reported by Li *et al.* [28]. The chemical shift for the compound **2** published by Li *et al*. [30] for OH (in *meta* position of ring A) δ = 14.21 is too high and δ = 9.63 for compound **9** is too small. This raises the possibility that compounds 2 and 9 prepared by Li *et al.* [28] are not identical to the denoted structures.

We found that the efficiency of the radical scavenging correlated with the position and number of hydroxyl groups in prepared compounds. Regarding the position of the hydroxyl group in hydroxyphenylimino parts of prepared compounds, most (hydroxyphenyliminomethyl)phenols with the OH group in *ortho* position of the ring A were the most potent scavengers of DPPH and galvinoxyl radicals. (Hydroxyphenyliminomethyl)phenols with three OH groups in the ring A were the most potent scavengers of ABTS radicals. (Hydroxyphenyliminomethyl)phenols **2**, **4**, **6**, **7**, **11**, **16** which contained one or two OH groups in positions 3 and 4 of the ring A exhibited relatively low ability to scavenge DPPH or GOR radicals (their values of SC_50_ ranged from 38 to 3075 μmol/dm^3^). However, compounds **3** and **5,** which have OH group in the *ortho* position of the benzene ring A, exhibited low antioxidant activity. On the other hand, compunds **17** and **21**, which do not have OH groups in the *ortho* position of the phenyl ring A, exhibited high antioxidant activity.

Based on the ^1^H-NMR spectra analyses, we assumed that proton could be most easily dissociation from the OH group in the *ortho* position of the ring A. (Hydroxyphenyliminomethyl)phenols with R^1^ = OH could form intramolecular hydrogen bond with the nitrogen of imine group. Due to this interaction acidity of hydrogen at this position increased, which was confirmed by the high chemical shift (δ = 12.25 to 14.19 ppm). On the other hand, the chemical shift of the OH protons bound in the *meta* or *para* position were <8.10 ppm.

It is generally assumed that the mechanism of radical scavenging by phenolic compounds is associated with the ability to release hydrogen atom, proton or electron from the antioxidant molecule. We speculated that releasing of the proton from OH group could be the main mechanism respossible for the antioxidant activity of studied (Hydroxyphenyliminomethyl)phenols. Consistent with this assumption were the SC_50_ values of studied compounds with higher δ which exhibited higher antioxidant activity as compared to other (Hydroxyphenyliminomethyl)phenols.

To find a possible correlation between the antioxidant activity of (hydroxyphenyliminomethyl)phenols and a position of the hydroxyl groups, we used the method PM6 (see the Experimental Section) to calculate the energy associated with the release of hydrogen (bond dissociation enthalpy, BDE); the proton relaxation (PDE); release of electron (IP ionization potential) and the energy associated with combined transfer of the electron and proton (ETE and PA, respectively) for the studied (Hydroxyphenyliminomethyl)phenols. The values of these energies in methanol or in water are presented in Tables S1–S8.

The antioxidant mechanism associated with the release of a hydrogen atom can be described by the Equation (1). From the point of view of thermodynamics the strength of the antioxidant effect in the model describes BDE (bond dissociation enthalpy, Equation (2)):

(1) ArOH + R^•^ → ArO^•^+ R^•^

(2) BDE = H(ArO^•^) + H(H^•^) − H(Ar-OH)

The other antioxidant mechanism of (hydroxyphenyliminomethyl)phenols may be associated with combined transfer of electrons and protons to radical (R^•^). In the first step, the electron is transferred to the radical (Equation (3)). The possibility of such transition is determined by the size of ionization potential of IP, which describes the Equation (4):

(3) ArOH + R^•^ → Ar-OH^•+^+ R^−^

(4) IP = H(Ar-OH^•+^) + H(e^−^) − H(Ar-OH)

In the second step, a proton is transferred to the anion radical R^−^ (Equation (5)). The possibility of such a transfer is characterized by the value of PDE (proton dissotiation enthalpy, Equation (6)):

(5) Ar-OH^•+^ + R^−^ → ArO^•^ + RH

(6) PDE = H(ArO^•^) + H(H^+^) − H(Ar-OH^•+^)

Another possible mechanism of the antioxidant effect of (hydroxyphenyliminomethyl)phenols is the gradual loss of a proton followed by electron transfer (Equations (7) and (8)):

(7) ArOH → ArO^−^ + H^+^

(8) ArO^−^ + R^•^ → ArO^•^ + R^−^

(9) R^−^ + H^+^ → RH

The reaction enthalpy of the first step (Equation (7)) corresponds to the proton affinity PA (Equation (10)) whereas the reaction enthalpy of the second step (Equation (8)) describes enthalpy of electron transfer (ETE, Equation (11)):

(10) PA = H(ArO^−^) + H(H^+^) − H(Ar-OH)

(11) ETE = H(ArO^•^) + H(e^−^) − H(ArO^−^)

**Figure 2:**
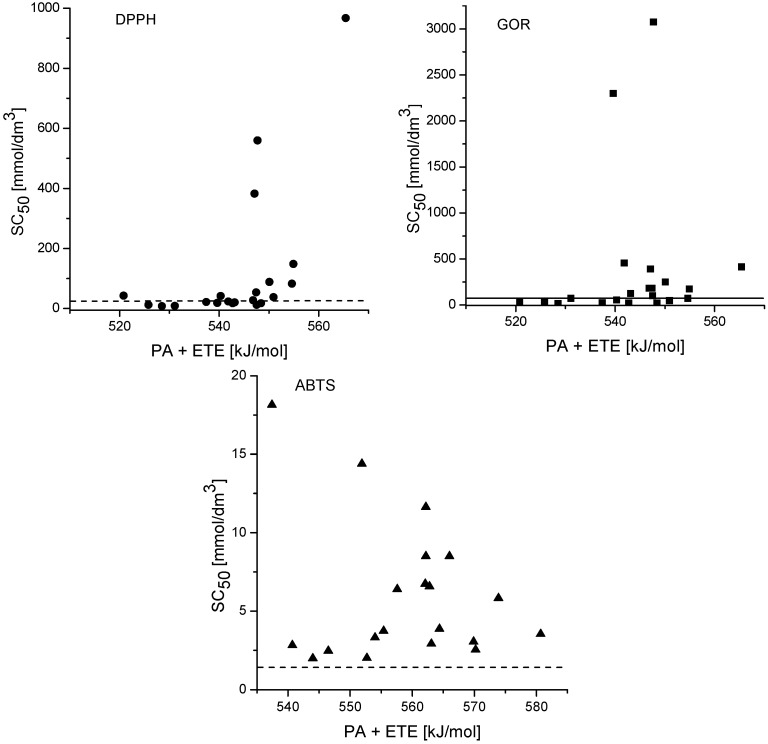
Dependence of SC_50_ of DPPH, GOR and ABTS scavenging by studied (Hydroxyphenyliminomethyl)phenols on PA + ETE entalpies. Dashed lines denote the SC_50_ values of resveratrol.

Comparison of enthalpies required for the above mentioned antioxidant reactions suggests that the preferred mechanism seems to be the one described by Equations (7)–(11), those characterized by the values of PA + ETE. Based on the above mentioned ideas, we speculated that the lower PA + ETE values of studied (hydroxyphenyliminomethyl)phenols may imply their higher antioxidant activity, *i.e.*, lower value of SC_50_. Figure 2 shows the dependence of SC_50_ scavenging DPPH radicals on the sum of PA + ETE enthalpy. As can be seen from these figures, there is no obvious relationship between these parameters. This discrepancy could be caused by forming of (hydroxyphenyliminomethyl)phenols self-aggregates which could reduce the antioxidant activity of original compounds.

**Figure 3:**
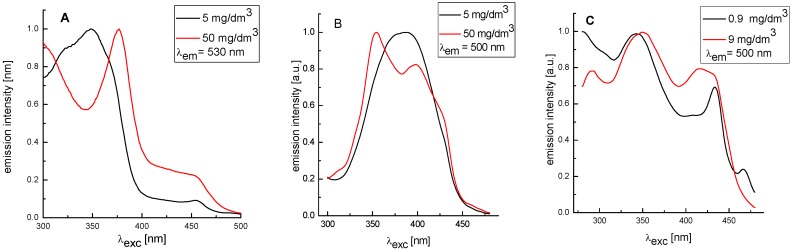
Fluorescence excitation spectra of studied (Hydroxyphenyliminomethyl)phenols: **5** (**A**); **14** (**B**) and **15** (**C**) in methanol at concentration of 0.9 mg/dm^3^. The excitation and emission slits were 2 resp. 10 nm.

To test this assumption we performed the following experiment. According to the work of Cigáň *et al.* [32], creation of self-aggregates can be determined from concentration-dependence of absorption or fluorescence spectra. The formation of self-aggregates should be manifested by a shift of emission maxima or by a shape change in absorption/fluorescence spectra with an increase of concentration of the studied compound. We observed that the corresponding changes in absorption spectra were very small. Also in fluorescence emission spectra, relatively small batochromic shifts were observed with increasing concentrations. Compound **5** with lower antioxidant activity exhibited approximately 11 nm, whereas compound **14** with higher antioxidant activity showed only 4 nm (data not shown). The formation of self-aggregates was apparent from the fluorescence excitation spectra. We observed changes in shapes of these spectra with the increasing concentrations of (Hydroxyphenyliminomethyl)phenols (Figure 3). These results suggest that one possible explanation for the lower antioxidant activity of some of the studied (Hydroxyphenyliminomethyl)phenols is formation of self-aggregates. A possible structure of aggregates for compound **3** is presented in Figure S1.

Numerous studies showed that various modifications of resveratrol molecule enhance its biological activities including the antioxidant properties [10,11,12,33]. It is therefore important to synthesize new resveratrol analogs and study their physico-chemical and biological properties. We aimed to identify novel analogues of resveratrol that could potently scavenge radicals and serve as leads for the development of future drugs. To find out more about potential pharmacological and nutritional relevance of (Hydroxyphenyliminomethyl)phenols synthesized in our current study, it will be important to test *in vivo* activities of these compounds. However, given their unstability in water, additional chemical modifications may be required to achieve high *in vivo* activity. Our current study brings new insights into the relationship between the chemical structure and antioxidant properties of resveratrol analogs.

## 3. Experimental Section

### 3.1. General Information

Methyl alcohol p.a. was purchased from Centralchem (Bratislava, Slovakia). 2-Aminophenol, 3-amino-phenol, 4-aminophenol, 2-hydroxybenzaldehyde, 3-hydroxybenzaldehyde, 4-hydroxy-benzaldehyde, 2,3-dihydroxybenzaldehyde, 2,4-dihydroxybenzaldehyde, 2,5-dihydroxybenzaldehyde, 3,4-dihydroxybenzaldehyde, 3,5-dihydroxybenzaldehyde, 2,3,4-trihydroxybenzaldehyde, 2,4,6-trihydroxybenzaldehyde, 3,4,5-trihydroxybenzaldehyde, 2,2-diphenyl-1-picrylhydrazyl, were purchased from Alfa Aesar (Heysham, UK). 2,2′-Azino-bis(3-ethylbenzothiazoline-6-sulfonic acid) diammonium salt (ABTS) was purchased from Sigma-Aldrich (St. Louis, MO, USA).

Absorption spectra were recorded by a Genesis 6 spectrophotometer (Thermo–Scientific, Waltham, MA, USA). Fluorescence emission spectra were recorded by a FSP 920 spectrofluorimeter (Edinburgh Instruments, Livingston, UK) and ^1^H- (300 MHz) and ^13^C-NMR (75 MHz) spectra were registered on a VNMRS 300 spectrometer (Varian, Salt Lake City, UT, USA) in DMSO-d_6_ with tetramethylsilane as internal standard. Melting points were determined on a Kofler hot plate apparatus and are uncorrected. Elemental analyses were obtained on an Elemental Analyzer CHNS-OEA 1108 (Carlo Erba, Wigan, UK). FTIR spectra (in solid phase) were recorded on a Nicolet 6700 spectrometer (Thermo–Scientific (Nicolet), Waltham, MA, USA) using the ATR technique.

MS spectra were recorded by a LC-MS spectrometer consisting of an Agilent 1200 HPLC (Walbron, Germany), with a MSD 6110 MS detector (Agilent Technologies, Santa Clara, CA, USA).

### 3.2. Synthesis

#### General Procedure for the Synthesis of (Hydroxyphenyliminomethyl)phenols

Twenty-one (hydroxyphenyliminomethyl)phenols were prepared following the protocol described by Tanaka and Shiraishi [16]. Briefly, equimolar amounts of mono-, di- and tri- hydroxybenzaldehydes (HBA) and 2-, 3- or 4-aminophenols (AP) were stirred (2 h) in distilled water at room temperature (25 °C). Obtained products were filtered, washed by water and dried at 45 °C. Yields of (Hydroxyphenyliminomethyl)phenols ranged between 36%–95%. The prepared compounds were characterized by melting point, elemental analysis, ^1^H- and ^13^C-NMR, IR and MS spectra.

*3-{[(2-Hydroxyphenyl)imino]methyl}phenol* (**1**): Yield 77%; m.p. 182–184 °C. Anal. Calc. for C_13_H_11_NO_2_ (213.24) C, 73.22; H, 5.20; N, 6.57. Found: C, 73.43; H, 5.04; N, 6.74%. IR: 1627.4 (*v*/C=N). ^1^H-NMR (DMSO-*d*_6_) δ: 13.77 (s, 1H), 9.71 (s, 1H), 8.96 (s, 1H), 7.61 (dd, *J* = 7.9, 1.3 Hz, 1H), 7.46–7.27 (m, 2H), 7.12 (ddd, 1H), 7.03–6.80 (m, 4H). ^13^C-NMR (DMSO-*d*_6_) δ: 162.10, 161.16, 151.56, 135.36, 133.26, 132.74, 128.49, 120.03, 120.00, 119.93, 119.16, 117.12, 116.95; positive LC-MS *m*/*z*: 214.1 [M + H]^+^ calc. for C_13_H_12_NO_2_^+^, 214.09, found 214.1

*2-{[(3-Hydroxyphenyl)imino]methyl}phenol* (**2**): Yield 71%; m.p. 116–118 °C. Anal. Calc. for C_13_H_11_NO_2_ (213.24) C, 73.22; H, 5.20; N, 6.57. Found: C, 73.47; H, 5.09; N, 6.50%. IR: 1619.9 (*v*/C=N).^1^H-NMR (DMSO-*d*_6_) δ: 9.64 (s, 1H), 8.97 (s, 1H), 8.58 (s, 1H), 7.41 (d, *J* = 7.8 Hz, 2H), 7.30 (dd, *J* = 7.7 Hz, 1H), 7.14 (d, *J* = 7.7 Hz, 1H), 7.07 (dd, *J* = 7.0 Hz, 1H), 7.00–6.73 (m, 3H).^13^C-NMR (DMSO-*d*_6_) δ: 160.04, 158.02, 151.35, 138.64, 138.16, 130.09, 127.58, 120.77, 119.89, 119.81, 118.91, 116.40, 115.05; positive LC-MS *m*/*z*: 214.1 [M + H]^+^ calc. for C_13_H_12_NO_2_^+^, 214.09, found 214.1.

*2-{[(3-Hydroxyphenyl)imino]methyl}phenol* (**3**): Yield 69%; m.p. 119–120 °C. Anal. Calc. for C_13_H_11_NO_2_ (213.24) C, 73.22; H, 5.20; N, 6.57. Found: C, 73.10; H, 5.13; N, 6.51%. IR: 1602.8 (*v/*C=N). ^1^H-NMR (DMSO-*d*_6_) δ: 13.12 (s, 1H), 9.64 (s, 1H), 8.90 (s, 1H), 7.65 (dd, *J* = 7.6, 1.5 Hz, 1H), 7.41 (ddd, *J* = 8.2, 1.6 Hz, 1H), 7.25 (dd, *J* = 7.9 Hz, 1H), 6.97 (dd, *J* = 8.3 Hz, 2H), 6.83 (d, *J* = 7.8 Hz, 1H), 6.77 (dd, *J* = 2.0 Hz, 1H), 6.73 (dd, *J* = 8.0, 1.7 Hz, 1H). ^13^C-NMR (DMSO-*d*_6_) δ: 163.64, 160.74, 158.75, 149.70, 133.65, 133.02, 130.62, 119.66, 119.52, 116.99, 114.51, 112.49, 108.56; positive LC-MS *m*/*z*: 214.1 [M + H]^+^ calc. for C_13_H_12_NO_2_^+^, 214.09, found 214.1.

*3-{[(4-Hydroxyphenyl)imino]methyl}phenol* (**4**): Yield 78%; m.p. 210–212 °C. Anal. Calc. for C_13_H_11_NO_2_ (213.24) C, 73.22; H, 5.20; N, 6.57. Found: C, 73.38; H, 5.23; N, 6.62%. IR: 1625.5 (*v*/C=N). ^1^H-NMR (DMSO-*d*_6_) δ: 10.09 (s, 1H), 9.44 (s, 1H), 8.39 (s, 1H), 7.75 (d, *J* = 8.4 Hz, 2H), 7.15 (t, *J* = 7.9 Hz, 1H), 6.87 (d, *J* = 8.4 Hz, 2H), 6.60 (dd, 3H). ^13^C-NMR (DMSO-*d*_6_) δ: 161.00, 160.01, 158.49, 153.82, 131.07, 130.22, 127.94, 116.07, 112.86, 112.02, 108.19; positive LC-MS *m/z*: 214.1 [M + H]^+^ calc. for C_13_H_12_NO_2_^+^, 214.09, found 214.1.

*2-{[(4-Hydroxyphenyl)imino]methyl}phenol* (**5**): Yield 38%; m.p. 138–139 °C. Anal. Calc. for C_13_H_11_NO_2_ (213.24) C, 73.22; H, 5.20; N, 6.57. Found: C, 73.40; H, 4.98; N, 6.49%. IR: 1614.3 (*v*/C=N). ^1^H-NMR (DMSO-*d*_6_) δ: 13.42 (s, 1H), 9.67 (s, 1H), 8.90 (s, 1H), 7.59 (dd, *J* = 7.6, 1.4 Hz, 1H), 7.47–7.21 (m, 3H), 7.08–6.89 (m, 2H), 6.86 (dd, *J* = 5.9 Hz, 2H). ^13^C-NMR (DMSO-*d*_6_) δ: 160.61, 160.58, 157.40, 139.61, 132.93, 132.63, 123.08, 119.86, 119.39, 116.89, 116.39; positive LC-MS *m*/*z*: 214.1 [M + H]^+^ calc. for C_13_H_12_NO_2_^+^, 214.09, found 214.1.

*3-{[(4-Hydroxyphenyl)imino]methyl}phenol* (**6**): Yield 84%; m.p. 172–173 °C. Anal. Calc. for C_13_H_11_NO_2_ (213.24) C, 73.22; H, 5.20; N, 6.57. Found: C, 73.04; H, 5.25; N, 6.41%. IR: 1622.4. (*v*/C=N),^1^H-NMR (DMSO-*d*_6_) δ: 9.62 (s, 1H), 9.48 (s, 1H), 8.51 (s, 1H), 7.41–7.23 (m, 3H), 7.18 (d, *J* = 8.7 Hz, 2H), 6.99–6.84 (m, 1H), 6.80 (d, *J* = 8.7 Hz, 2H). ^13^C-NMR (DMSO-*d*_6_) δ: 157.67, 157.18, 156.27, 142.59, 137.86, 129.78, 122.51, 119.99, 118.19, 115.74, 113.89; positive LC-MS *m*/*z*: 214.1 [M + H]^+^ calc. for C_13_H_12_NO_2_^+^, 214.09, found 214.1.

*4-{[(4-Hydroxyphenyl)imino]methyl}phenol* (**7**): Yield 82%; m.p. 183–185 °C. Anal. Calc. for C_13_H_11_NO_2_ (213.24) C, 73.22; H, 5.20; N, 6.57. Found: C, 73.48; H, 5.02; N, 6.33%. IR: 1639.6 (*v*/C=N). ^1^H-NMR (DMSO-*d*_6_) δ: 10.00 (s, 1H), 9.37 (s, 1H), 8.44 (s, 1H), 7.72 (d, *J* = 8.6 Hz, 2H), 7.11 (d, *J* = 8.7 Hz, 2H), 6.86 (d, *J* = 8.6 Hz, 2H), 6.77 (d, *J* = 8.7 Hz, 2H). ^13^C-NMR (DMSO-*d*_6_) δ: 160.10, 156.89, 155.68, 143.21, 130.18, 127.91, 122.15, 115.64, 115.58; positive LC-MS *m/z*: 214.1 [M + H]^+^ calc. for C_13_H_12_NO_2_^+^, 214.09, found 214.1.

*3-{[(2-Hydroxyphenyl)imino]methyl}benzene-1,2-diol* (**8**): Yield 75%; m.p. 168–169 °C. Anal. Calc. for C_13_H_11_NO_3_ (229.24) C, 68.11; H, 4.84; N, 6.11. Found: C, 68.29; H, 5.06; N, 6.14 %. IR: 1627.7 (*v*/C=N). ^1^H-NMR (DMSO-*d*_6_) δ: 14.19 (s, 1H), 9.83 (s, 1H), 9.02 (s, 1H), 8.94 (s, 1H), 7.41 (dd, *J* = 7.8, 1.5 Hz, 1H), 7.13 (ddd, *J* = 7.8, 1.5 Hz, 1H), 7.04 (dd, *J* = 7.8, 1.5 Hz, 1H), 6.98 (dd, *J* = 8.1, 1.2 Hz, 1H), 6.94–6.85 (m, 2H), 6.72 (dd, *J* = 7.8 Hz, 1H). ^13^C-NMR (DMSO-*d*_6_) δ: 161.63, 152.02, 151.29, 146.49, 134.38, 128.40, 122.89, 120.06, 119.60, 119.41, 118.47, 118.30, 116.91; positive LC-MS *m/z*: 230.1 [M + H]^+^ calc. for C_13_H_12_NO_3_^+^, 230.08, found 230.1.

*4-{[(2-Hydroxyphenyl)imino]methyl}benzene-1,3-diol* (**9**): Yield 77%; m.p. 152–155 °C Anal. Calc. for C_13_H_11_NO_3_ (229.24) C, 68.11; H, 4.84; N, 6.11. Found: C, 68.29; H, 5.06; N, 6.14%. IR: 1627.7 (*v*/C=N), ^1^H-NMR (DMSO-*d*_6_) δ: 14.20 (s, 1H), 10.13 (s, 1H), 9.62 (s, 1H), 8.76 (s, 1H), 7.35 (d, *J* = 8.6 Hz, 1H), 7.28 (dd, *J* = 7.9, 1.5 Hz, 1H), 7.10–7.01 (m, 1H), 6.91 (dd, *J* = 8.0, 1.3 Hz, 1H), 6.83 (td, *J* = 7.8, 1.4 Hz, 1H), 6.32 (dd, *J* = 8.5, 2.3 Hz, 1H), 6.21 (d, *J* = 2.3 Hz, 1H). ^13^C-NMR (DMSO-*d*_6_) δ: 165.40, 162.90, 160.66, 150.90, 134.91, 134.62, 127.52, 120.03, 119.47, 116.75, 112.66, 107.94, 103.09; positive LC-MS *m*/*z*: 230.0 [M + H]^+^ calc. for C_13_H_12_NO_3_^+^, 230.08, found 230.0.

*2-{[(2-Hydroxyphenyl)imino]methyl}benzene-1,4-diol* (**10**): Yield 71%; m.p. 135–137 °C. Anal. Calc. for C_13_H_11_NO_3_ (229.24) C, 68.11; H, 4.84; N, 6.11. Found: C, 67.92; H, 4.72; N, 5.88 %. IR: 1638.5 (*v*/C=N), ^1^H-NMR (DMSO-*d*_6_) δ: 12.86 (s, 1H), 9.63 (s, 1H), 9.03 (s, 1H), 8.83 (s, 1H), 7.32 (dd, *J* = 7.9, 1.5 Hz, 1H), 7.16–7.05 (m, 1H), 7.03–6.72 (m, 5H). ^13^C-NMR (DMSO-*d*_6_) δ: 162.02, 153.79, 151.48, 149.80, 135.91, 128.28, 121.09, 120.05, 120.01, 119.90, 117.56, 117.31, 116.90; positive LC-MS *m*/*z*: 230.1 [M + H]^+^ calc. for C_13_H_12_NO_3_^+^, 230.08, found 230.1.

*5-{[(2-Hydroxyphenyl)imino]methyl}benzene-1,3-diol* (**11**): Yield 26%; m.p. 174–175 °C. Anal. Calc. for C_13_H_11_NO_3_ (229.24) C, 68.11; H, 4.84; N, 6.11. Found: C, 68.32; H, 4.69; N, 5.86%. IR: 1628.1 (*v*/C=N). ^1^H-NMR (DMSO-*d*_6_): δ = 9.45 (s, 2H), 8.98 (s, 1H), 8.44 (s, 1H), 7.10 (dd, *J* = 7.8, 1.5 Hz, 1H), 7.05 (ddd, *J* = 7.8, 7.7, 1.5, 1H), 6.87 (dd, J = 8.0, 1.3 Hz, 1H), 6.85 (d, *J* = 2.2 Hz, 2H), 6.81 (ddd, *J* = 7.7, 7.4, 1.3 Hz, 1H), 6.36 (dd, *J* = 2.2 Hz, 1H). ^13^C-NMR (DMSO-*d*_6_): δ = 160.40, 158.97, 151.18, 138.87, 138.64, 127.40, 119.98, 119.88, 116.35, 107.30, 105.99; positive LC-MS *m*/*z*: 230.1 [M + H]^+^ calc. for C_13_H_12_NO_3_^+^, 230.08, found 230.1.

*3-{[(3-Hydroxyphenyl)imino]methyl}benzene-1,2-diol* (**12**): Yield 79%; m.p. 184–185 °C. Anal. Calc. for C_13_H_11_NO_3_ (229.24) C, 68.11; H, 4.84; N, 6.11. Found: C, 68.32; H, 4.65; N, 5.87%. IR: 1632.9 (*v*/C=N), ^1^H-NMR (DMSO-*d*_6_) δ: 13.25 (s, 1H), 9.64 (s, 1H), 9.18 (s, 1H), 8.86 (s, 1H), 7.25 (dd, *J* = 7.9 Hz, 1H), 7.09 (dd, *J* = 7.8, 1.4 Hz, 1H), 6.94 (dd, *J* = 7.8, 1.4 Hz, 1H), 6.88–6.67 (m, 4H). ^13^C-NMR (DMSO-*d*_6_) δ: 163.57, 158.34, 149.48, 149.01, 145.62, 130.24, 122.81, 119.28, 118.91, 118.72, 114.07, 111.97, 108.13; positive LC-MS *m*/*z*: 230.1 [M + H]^+^ calc. for C_13_H_12_NO_3_^+^, 230.08, found 230.1.

*2-{[(3-Hydroxyphenyl)imino]methyl}benzene-1,4-diol* (**13**): Yield 89%; m.p. 175–177 °C. Anal. Calc. for C_13_H_11_NO_3_ (229.24) C, 68.11; H, 4.84; N, 6.11. Found: 68.30; H, 4.73; N, 5.91%. IR: 1634.1 (*v*/C=N), ^1^H-NMR (DMSO-*d*_6_) δ: 12.27 (s, 1H), 9.62 (s, 1H), 9.08 (s, 1H), 8.78 (s, 1H), 7.23 (dd, *J* = 7.9 Hz, 1H), 7.03 (d, *J* = 2.9 Hz, 1H), 6.91–6.64 (m, 5H). ^13^C-NMR (DMSO-*d*_6_) δ: 163.29, 158.70, 153.50, 150.11, 150.02, 130.56, 121.47, 119.65, 117.55, 117.38, 114.32, 112.46, 108.53; positive LC-MS *m*/*z*: 230.1 [M + H]^+^ calc. for C_13_H_12_NO_3_^+^, 230.08, found 230.1.

*4-{[(4-Hydroxyphenyl)imino]methyl}benzene-1,3-diol* (**14**): Yield 55%; m.p. 130–132 °C. Anal. Calc. for C_13_H_11_NO_3_ (229.24) C, 68.11; H, 4.84; N, 6.11. Found: 68.27; H, 5.06; N, 5.90%. IR: 1617.4 (*v*/C=N). ^1^H-NMR (DMSO-*d*_6_) δ: 13.80 (s, 1H), 10.13 (s, 1H), 9.55 (s, 1H), 8.72 (s, 1H), 7.37 (d, *J* = 8.5 Hz, 1H), 7.29–7.16 (m, 2H), 6.86–6.76 (m, 2H), 6.38 (dd, *J* = 8.4, 2.3 Hz, 1H), 6.27 (d, *J* = 2.3 Hz, 1H). ^13^C-NMR (DMSO-*d*_6_) δ: 163.19, 162.23, 160.18, 156.67, 139.86, 134.33, 122.57, 116.31, 112.59, 107.98, 102.79; positive LC-MS *m*/*z*: 230.1 [M + H]^+^ calc. for C_13_H_12_NO_3_^+^, 230.08, found 230.1.

*2-{[(4-Hydroxyphenyl)imino]methyl}benzene-1,4-diol* (**15**): Yield 92%; m.p. 196–197 °C. Anal. Calc. for C_13_H_11_NO_3_ (229.24) C, 68.11; H, 4.84; N, 6.11. Found: 67.92; H, 4.97; N, 6.26%. IR: 1624.5 (*v*/C=N). ^1^H-NMR (DMSO-*d*_6_) δ: 12.58 (s, 1H), 9.64 (s, 1H), 9.04 (s, 1H), 8.79 (s, 1H), 7.29 (d, *J* = 8.7 Hz, 2H), 6.98 (d, *J* = 2.7 Hz, 1H), 6.93–6.52 (m, 4H). ^13^C-NMR (DMSO-*d*_6_) δ: 160.39, 157.26, 153.35, 149.94, 139.99, 123.05, 120.70, 119.82, 117.40, 117.27, 116.35; positive LC-MS *m*/*z*: 230.1 [M + H]^+^ calc. for C_13_H_12_NO_3_^+^, 230.08, found 230.1.

*5-{[(4-Hydroxyphenyl)imino]methyl}benzene-1,3-diol* (**16**): Yield 92%; m.p. 118–120 °C. Anal. Calc. for C_13_H_11_NO_3_ (229.24) C, 68.11; H, 4.84; N, 6.11. Found: 67.92; H, 4.97; N, 6.26%. IR: 1620.5 (*v*/C=N). ^1^H-NMR (DMSO-*d*_6_) δ: 9.44 (s, 3H), 8.38 (s, 1H), 7.15 (d, *J* = 8.7 Hz, 2H), 6.87–6.69 (m, 4H), 6.32 (dd, *J* = 2.1 Hz, 1H). ^13^C-NMR (DMSO-*d*_6_) δ: 159.05, 157.85, 156.61, 143.02, 138.78, 122.90, 116.12, 106.80, 105.66; positive LC-MS *m*/*z*: 230.1 [M + H]^+^ calc. for C_13_H_12_NO_3_^+^, 230.08, found 230.1.

*4-{[(3-Hydroxyphenyl)imino]methyl}benzene-1,2,3-triol* (**17**): Yield 89%; m.p. 254–256 °C. Anal. Calc. for C_13_H_11_NO_4_ (245.24) C, 63.67; H, 4.52; N, 5.71. Found: 63.96; H, 4.48; N, 5.50%. IR: 1606.2 (*v*/C=N). ^1^H-NMR (DMSO-*d*_6_) δ: 13.70 (s, 1H), 9.63 (d, 2H), 8.72 (s, 1H), 8.43 (s, 1H), 7.22 (dd, *J* = 7.9 Hz, 1H), 6.94 (d, *J* = 8.6 Hz, 1H), 6.78 (d, *J* = 8.9 Hz, 1H), 6.76–6.62 (m, 2H), 6.40 (d, *J* = 8.5 Hz, 1H). ^13^C-NMR (DMSO-*d*_6_) δ: 163.25, 158.96, 152.61, 150.89, 149.38, 133.05, 130.83, 124.74, 114.04, 112.82, 112.23, 108.41; positive LC-MS *m*/*z*: 246.1 [M + H]^+^, 268.0 [M + Na]^+^. Calc. for C_13_H_12_NO_3_^+^, 246.08, found 246.1.

*2-{[(3-Hydroxyphenyl)imino]methyl}benzene-1,3,5-triol* (**18**): Yield 94%; m.p. 257–259 °C. Anal. Calc. for C_13_H_11_NO_4_ (245.24) C, 63.67; H, 4.52; N, 5.71. Found: 63.87; H, 4.39; N, 5.78%. IR: 1626.1 (*v*/C=N). ^1^H-NMR (DMSO-*d*_6_) δ: 12.25 (s, 2H), 10.07 (s, 1H), 9.56 (s, 1H), 8.87 (s, 1H), 7.20 (dd, *J* = 11.1, 5.5 Hz, 1H), 6.68 (dd, *J* = 18.1, 7.3 Hz, 3H), 5.81 (s, 2H). ^13^C-NMR (DMSO-*d*_6_) δ: 163.91, 162.62, 158.33, 157.10, 149.27, 130.28, 113.11, 111.57, 107.33, 101.46, 94.10; positive LC-MS *m*/*z*: 246.1 [M + H]^+^, calc. for C_13_H_12_NO_3_^+^, 246.08, found 246.1.

*5-{[(3-Hydroxyphenyl)imino]methyl}benzene-1,2,3-triol* (**19**): Yield 47%; m.p. 283–285 °C. Anal. Calc. for C_13_H_11_NO_4_ (245.24) C, 63.67; H, 4.52; N, 5.71. Found: 63.77; H, 4.39; N, 5.78%. IR: 1644.2 (*v*/C=N). ^1^H-NMR (DMSO-*d*_6_) δ: 9.42 (s, 1H), 9.17 (s, 2H), 8.76 (s, 1H), 8.22 (s, 1H), 7.14 (dd, *J* = 7.9 Hz, 1H), 6.87 (s, 2H), 6.70–6.48 (m, 3H)m. ^13^C-NMR (DMSO-*d*_6_) δ: 192.00, 160.66, 158.66, 153.92, 150.43, 146.78, 146.68, 140.86, 137.71, 130.43, 130.07, 128.10, 127.40, 112.97, 112.21, 109.41, 108.56, 108.35, 106.07, 103.94, 101.60; positive LC-MS *m*/*z*: 246.1 [M + H]^+^, 268.1 [M + Na]^+^. Calc. for C_13_H_12_NO_3_^+^, 246.08, found 246.1.

*2-{[(4-Hydroxyphenyl)imino]methyl}benzene-1,3,5-triol* (**20**): Yield 37%; m.p. 173–175 °C. Anal. Calc. for C_13_H_11_NO_4_ (245.24) C, 63.67; H, 4.52; N, 5.71. Found: C, 63.76; H, 4.69; N, 5.88%. IR: 1616.0 (*v*/C=N). ^1^H-NMR (DMSO-*d*_6_) δ: 12.27 (s, 2H), 9.94 (s, 1H), 9.48 (s, 1H), 8.85 (s, 1H), 7.14 (d, *J* = 8.7 Hz, 2H), 6.79 (d, *J* = 8.7 Hz, 2H), 5.79 (s, 2H). ^13^C-NMR (DMSO-*d*_6_) δ: 163.53, 156.34, 155.39, 140.10, 122.21, 116.39, 101.85, 94.44; positive LC-MS *m*/*z*: 246.1 [M + H]^+^, calc. for C_13_H_12_NO_3_^+^, 246.08, found 246.1.

*5-{[(4-Hydroxyphenyl)imino]methyl}benzene-1,2,3-triol* (**21**): Yield 89%; m.p. 228–229 °C. Anal. Calc. for C_13_H_11_NO_4_ (245.24) C, 63.67; H, 4.52; N, 5.71. Found: C, 63.80; H, 4.41; N, 5.87%. IR: 1640.0 (*v*/C=N). ^1^H-NMR (DMSO-*d*_6_) δ: 9.35 (s, 1H), 9.11 (s, 2H), 8.59 (d, 1H), 8.26 (s, 1H), 7.23–7.00 (m, 2H), 6.85 (d, *J* = 2.8 Hz, 2H), 6.81–6.67 (m, 2H). ^13^C-NMR (DMSO-*d*_6_) δ: 168.82, 148.74, 145.74, 140.95, 136.83, 124.97, 116.02, 115.85, 107.57; positive LC-MS *m*/*z*: 246.1 [M + H]^+^, 268.0 [M + Na]^+^. Calc. for C_13_H_12_NO_3_^+^, 246.08, found 246.1.

### 3.3. DPPH Assay

Scavenging of DPPH radicals by prepared (hydroxyphenyliminomethyl)phenols was performed according to our previous work Šeršeň *et al.* [34]. Briefly, various amounts of tested (Hydroxyphenyliminomethyl)phenols were added into methanol solution of DPPH and the final DPPH concentration was kept constant (*c* = 10^−4^ mol·dm^−3^). 20 min after the addition of the tested substance, absorbance was measured at 517 nm. The antioxidant activity was evaluated using the values SC_50_, *i.e.*, concentration of the studied compound, which causes a 50% decrease in absorbance at 517 nm as compared to the control sample. Methanol was used as a blank.

### 3.4. Scavenging of Galvinoxyl Radicals

Various amounts of (hydroxyphenyliminomethyl)phenols were added into methanol solution of galvinoxyl radical such that the final galvinoxyl concentration was *c* = 10^−4^ mol·dm^−3^. 20 min after the addition of (hydroxyphenyliminomethyl)phenols, absorbance was measured at 862 nm. SC_50_ values were calculated from the absorbance *vs.* the concentration dependence. Methanol was used as a blank.

### 3.5. ABTS Assay

ABTS cation radicals were prepared according to Kurin *et al.* [35] with minor adaptations. Briefly 7 mM aqueous solution of ABTS was mixed in equimolar ratio with 2.45 mM K_2_S_2_O_8_, and let 24 h to stand in the dark at room temperature. After 24 h 1.1 mL of concentrated solution of the radical was diluted with 50 mL of phosphate buffer (solution A: 900 mg of Na_2_HPO_4_ in 500 mL H_2_O, the solution B: 340 mg of KH_2_HPO_4_ in 500 mL H_2_O, we combined 9 parts of solution A and 1 part of solution B, the pH we set to 7.4). To a solution of 1.8 mL of ABTS we added 0, 25, 50, 75, 100, 125, 150, 175 or 200 mL of the test substance and completed with 200, 175, 150, 125, 100, 75, 50, 25 or 0 mL of ethanol (to a final volume of 2 mL. The absorbance at 734 nm of prepared solutions was measured after 6 min. We calculated the appropriate values of the SC_50_ from the linear part of the absorbance *vs.* the concentration of (hydroxyphenyliminomethyl)phenols. The water was used as blank.

### 3.6. Molecular Calculations

The prepared (hydroxyphenyliminomethyl)phenols, their anions and radicals were studied using the quantum chemical method PM6 [36], which is part of the program MOPAC2012 [37]. Optimal structures of compunds were calculated (keyword PRECISE). The effect of solvents on the above mentioned compounds were studied by COSMO-method [38], which is also part of the MOPAC2012 [39]. Ionization potentials and heats of formations used for the calculation of PDE, BDE, PA and ETE according to the Equations (2), (6), (10) and (11) were obtained using the method PM6.

## 4. Conclusions

In this work we prepared twenty-one (hydroxyphenyliminomethyl)phenols, which were synthesized by condensation reactions of the corresponding aminophenols and hydroxybenzaldehydes. The chemical structures of these compounds were confirmed by ^1^H-NMR, IR spectroscopy, elemental analysis and GCMS. All prepared (hydroxyphenyliminomethyl)phenols were able to scavenge DPPH, GOR and ABTS radicals. Some of the (hydroxyphenyliminomethyl)phenols were more potent scavengers of DPPH (compounds: **8**, **10**, **12**–**14**, **11**–**27**) and GOR (compounds: **2**, **9**, **12**, **15**, **17**–**21**) radicals as compared to resveratrol. The efficiency of antioxidant activity correlated with the number and position of hydroxyl groups. The most efficient antioxidants were (hydroxyphenyliminomethyl)phenols containing three hydroxyl groups in the benzylidene part of molecules.

## Supplementary Materials

Supplementary materials can be accessed at: http://www.mdpi.com/1420-3049/21/1/127/s1.

## Abbreviations

The following abbreviations are used in this manuscript:

ABTS: 2,2′-azino-bis(3-ethylbenzothiazoline)-6-sulphonic acid
AP: aminophenol
BDE: bond dissociation enthalpy
DPPH: 2,2-diphenyl-1-picrylhydrazyl
ETE: electron transfer enthalpy
GOR: galvinoxyl radical
HBA: hydroxybenzaldehyde
IP: ionization potential
IR: infrared
MS: mass spectrometry
NMR: nuclear magnetic resonance
PA: proton affinity
