# Supplementary material for: The Scavenging of DPPH, Galvinoxyl and ABTS Radicals by Imine Analogs of Resveratrol
Source: Molecules. 2016 Jan 21;21(1):127. doi: 10.3390/molecules21010127 (PMC4759939; doi:10.3390/molecules21010127)
Supplement: Supplementary file 1 [file molecules-21-00127-s001.pdf]

Supplementary Materials: The Scavenging of DPPH, Galvinoxyl and ABTS Radicals by Imine Analogs of Resveratrol

Peter Kotora [1,*], František Šeršeň [1], Juraj Filo [1], Dušan Loos [1], Juraj Gregáň [2,3,*] and Fridrich Gregáň [4]

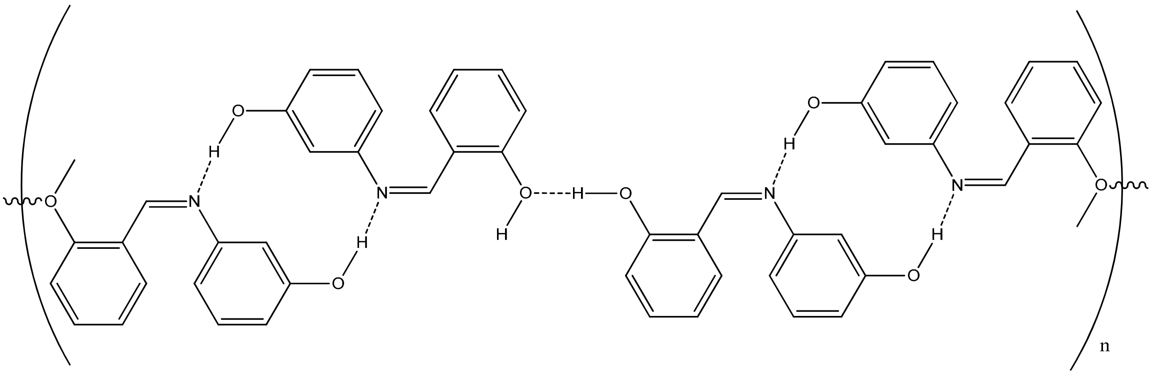

Scheme S1. A possible structure of aggregates for compound **3**.

Table S1. Proton dissociation energy of prepared (hydroxyphenyliminomethyl)phenols in methanol.

	PDE (kJ/mol)					
**Compound**	**H⁺ from R⁶⁻⁸**	**H⁺ from R¹**	**H⁺ from R²**	**H⁺ from R³**	**H⁺ from R⁴**	**H⁺ from R⁵**
**1**	201.4	225.0				
**2**	151.9		163.8			
**3**	130.9	149.4				
**4**	170.7			170.2		
**5**	202.0	231.0				
**6**	167.9		183.1			
**7**	174.7			182.9		
**8**	220.2	214.1	215.2			
**9**	166.4	225.9		217.2		
**10**	220.4	215.2			219.3	
**11**	149.4		165.1		164.6	
**12**	149.0	120.7	123.4			
**13**	228.4	205.8			211.0	
**14**	177.9	189.8		192.4		
**15**	220.7	216.0			220.2	
**16**	167.5		190.6		185.8	
**17**	144.4	124.3	116.2	124.1		
**18**	152.4	112.1		181.0		196.0
**19**	162.8		141.9	127.6	142.1	
**20**	155.8	186.3		190.5		186.5
**21**	161.1		161.3	144.7	161.5	
resveratrol	143.7		163.1		163.3	

Table S2. Dissociation energy of hydrogen and electron of prepared (hydroxyphenyliminomethyl) phenols in methanol.

	BDE (kJ/mol)						IP (kJ/mol)
**Compound**	**H⁺ from $R^{6–8}$**	**H⁺ from R^1**	**H⁺ from R^2**	**H⁺ from R^3**	**H⁺ from R^4**	**H⁺ from R^5**	
**1**	316.7	340.3					859.6
**2**	320.8		332.6				874.4
**3**	317.6	336.1					888.0
**4**	335.9			335.3			886.9
**5**	317.0	345.9					850.0
**6**	320.0		335.2				864.8
**7**	317.3			325.6			850.4
**8**	313.1	306.9	308.0				871.8
**9**	290.7	350.2		341.6			853.0
**10**	314.8	309.6			313.7		844.5
**11**	324.7		340.5		340.0		882.5
**12**	337.3	309.0	311.7				880.7
**13**	335.3	312.6			317.9		860.4
**14**	317.4	329.3		332.0			848.9
**15**	314.9	310.2			314.4		845.4
**16**	324.8		347.9		343.1		873.5
**17**	335.6	315.4	307.3	315.3			887.4
**18**	336.0	295.7		364.6		379.6	889.6
**19**	336.2		315.3	301.0	315.5		890.1
**20**	318.3	348.7		352.9		348.9	855.0
**21**	314.7		314.9	298.3	315.1		858.3
resveratrol	318.5		337.8		338.0		868.3

Table S3. Proton affinity of prepared (hydroxyphenyliminomethyl)phenols in methanol.

	PA (kJ/mol)					
**Compound**	**H⁺ from $R^{6–8}$**	**H⁺ from R^1**	**H⁺ from R^2**	**H⁺ from R^3**	**H⁺ from R^4**	**H⁺ from R^5**
**1**	214.1	264.4				
**2**	270.2		262.8			
**3**	223.7	249.7				
**4**	261.5			255.4		
**5**	224.0	281.6				
**6**	263.1		262.3			
**7**	264.6			254.4		
**8**	205.2	262.3	257.0			
**9**	205.6	246.2		242.5		
**10**	216.4	280.1			266.8	
**11**	269.6		249.0		249.7	
**12**	218.6	254.1	265.0			
**13**	259.7	268.3			266.9	
**14**	266.1	237.9		240.4		
**15**	258.9	255.1			267.9	
**16**	264.2		252.9		250.2	
**17**	211.0	229.7	256.4	239.3		
**18**	212.1	228.8		235.7		253.8
**19**	260.0		239.2	246.8	239.3	
**20**	211.9	230.4		236.3		230.4
**21**	262.0		240.1	248.3	239.6	
resveratrol	261.5		253.0		253.1	

Table S4. Electron transfer enthalpy of prepared (hydroxyphenyliminomethyl)phenols in methanol.

	ETE (kJ/mol)					
**Compound**	**H^+ from $R^{6–8}$**	**H^+ from R^1**	**H^+ from R^2**	**H^+ from R^3**	**H^+ from R^4**	**H^+ from R^5**
**1**	332.7	306.0				
**2**	280.7		299.9			
**3**	324.0	316.6				
**4**	304.5			310.0		
**5**	323.1	294.5				
**6**	287.0		303.0			
**7**	282.8			301.3		
**8**	337.9	274.7	281.1			
**9**	315.2	334.2		329.2		
**10**	328.5	259.6			277.0	
**11**	284.6		321.0		319.8	
**12**	348.8	285.0	276.8			
**13**	305.7	274.4			281.1	
**14**	281.4	321.5		321.7		
**15**	286.1	285.2			276.6	
**16**	290.7		325.2		323.0	
**17**	354.7	315.8	281.0	306.0		
**18**	354.1	297.0		359.1		355.9
**19**	306.3		306.3	284.3	306.3	
**20**	336.5	348.4		346.7		348.6
**21**	282.9		304.9	280.2	305.6	
resveratrol	287.1		315.0		315.0	

Table S5. Proton dissociation energy of prepared (hydroxyphenyliminomethyl)phenols in water.

	PDE (kJ/mol)					
**Compound**	**H^+ from $R^{6–8}$**	**H^+ from R^1**	**H^+ from R^2**	**H^+ from R^3**	**H^+ from R^4**	**H^+ from R^5**
**1**	160.4	183.5				
**2**	111.1		87.4			
**3**	89.3	108.3				
**4**	129.3			128.8		
**5**	160.9	189.3				
**6**	126.8		142.0			
**7**	133.2			141.5		
**8**	179.5	173.0	173.7			
**9**	123.8	180.3		170.5		
**10**	179.2	173.6			178.0	
**11**	108.4		124.1		123.7	
**12**	108.2	80.0	82.7			
**13**	189.6	166.7			172.3	
**14**	136.0	148.1		150.7		
**15**	179.6	174.8			179.2	
**16**	126.0		145.0		144.4	
**17**	103.4	83.2	75.0	82.5		
**18**	111.0	70.5		139.5		153.9
**19**	121.6		101.0	86.8	101.1	
**20**	114.3	145.1		114.4		145.2
**21**	120.0		120.3	103.9	120.6	
resveratrol	102.9		122.4		122.6	

Table S6. Dissociation energy of hydrogen and electron of prepared (hydroxyphenyliminomethyl) phenols in water.

Compound	BDE (kJ/mol)						IP (kJ/mol)
	H^+ from $R^{6–8}$	H^+ from R^1	H^+ from R^2	H^+ from R^3	H^+ from R^4	H^+ from R^5	
**1**	320.6	331.9					860.5
**2**	316.8		340.0				875.2
**3**	316.9	335.9					888.7
**4**	335.8			335.4			887.7
**5**	316.8	345.1					850.6
**6**	319.1		334.3				864.5
**7**	317.3			325.6			851.2
**8**	313.0	306.6	307.2				872.2
**9**	289.3	345.8		336.6			853.7
**10**	314.9	309.3			313.7		845.6
**11**	324.6		340.1		339.9		883.4
**12**	337.0	308.7	311.4				881.4
**13**	335.1	312.3			317.8		861.0
**14**	316.5	328.6		331.1			850.0
**15**	314.7	310.0			314.4		846.1
**16**	324.8		343.8		343.2		874.2
**17**	335.8	315.6	307.4	314.9			887.9
**18**	335.9	295.4		364.3		378.7	890.4
**19**	336.0		315.4	301.2	315.5		890.6
**20**	317.7	348.4		317.7		348.6	855.8
**21**	314.7		315.0	298.6	315.2		858.9
resveratrol	318.2		337.6		337.8		868.7

Table S7. Proton affinity of prepared (hydroxyphenyliminomethyl)phenols in water.

	PA (kJ/mol)					
**Compound**	**H^+ from $R^{6–8}$**	**H^+ from R^1**	**H^+ from R^2**	**H^+ from R^3**	**H^+ from R^4**	**H^+ from R^5**
**1**	163.2	213.1				
**2**	217.7		210.7			
**3**	172.1	198.4				
**4**	209.2			204.3		
**5**	172.5	229.0				
**6**	210.8		209.1			
**7**	213.2			203.0		
**8**	154.6	211.2	212.4			
**9**	154.8	194.3		191.5		
**10**	165.7	211.6			214.9	
**11**	218.0		198.0		198.8	
**12**	167.1	202.2	213.0			
**13**	208.0	200.4			215.2	
**14**	213.4	186.1		188.5		
**15**	207.3	203.7			215.9	
**16**	213.5		197.6		198.5	
**17**	160.3	179.5	205.7	187,.8		
**18**	160.4	178.3		184.8		201.4
**19**	208.8		188.1	196.2	187.7	
**20**	16.1	179.3		160.1		179.2
**21**	211.1		188.8	197.4	188.1	
resveratrol	210.4		201.0		201.1	

Table S8. Electron transfer enthalpy of prepared (hydroxyphenyliminomethyl)phenols in water.

	ETE (kJ/mol)					
**Compound**	**H^+ from R^{6-8}**	**H^+ from R^1**	**H^+ from R^2**	**H^+ from R^3**	**H^+ from R^4**	**H^+ from R^5**
**1**	399.0	372.2				
**2**	348.3		366.5			
**3**	390.1	382.9				
**4**	371.9			376.4		
**5**	389.6	361.5				
**6**	353.6		370.6			
**7**	349.5			367.9		
**8**	403.7	340.7	340.2			
**9**	379.9	396.9		389.9		
**10**	394.6	343.1			344.2	
**11**	351.9		387.7		386.5	
**12**	415.2	351.8	343.8			
**13**	372.5	357.2			348.0	
**14**	348.5	387.8		388.0		
**15**	352.8	351.7			343.8	
**16**	356.7		391.6		390.0	
**17**	420.9	381.5	347.0	372.4		
**18**	420.8	362.4		424.9		422.7
**19**	373.4		372.6	350.3	373.1	
**20**	403.0	414.5		403.0		414.7
**21**	348.9		371.6	346.6	372.5	
resveratrol	353.1		382.0		382.1	
